# Comprehensive Analysis of a circRNA-miRNA-mRNA Network to Reveal Potential Inflammation-Related Targets for Gastric Adenocarcinoma

**DOI:** 10.1155/2020/9435608

**Published:** 2020-08-01

**Authors:** YunXia Liu, YeFeng Xu, Feng Xiao, JianFeng Zhang, YiQing Wang, YongWei Yao, JieWen Yang

**Affiliations:** ^1^Department of Oncology, Hangzhou Third Hospital, Hangzhou, Zhejiang, China; ^2^Affiliated Hangzhou Clinical College, Anhui Medical University, Hangzhou, Zhejiang, China; ^3^Zhejiang Chinese Medical University, Hangzhou, Zhejiang, China

## Abstract

Gastric cancer (GC) is the most common malignancy of the stomach. This study was aimed at elucidating the regulatory network of circRNA-miRNA-mRNA and identifying the precise inflammation-related targets in GC. The expression profiles of GSE83521, GSE78091, and GSE33651 were obtained from the GEO database. Interactions between miRNAs and circRNAs were investigated by the Circular RNA Interactome, and targets of miRNAs were predicted with miRTarBase. Then, a circRNA/miRNA/mRNA regulatory network was constructed. Also, functional enrichment analysis of selected differentially expressed genes (DEGs) was performed. The inflammation-/GC-related targets were collected in the GeneCards and GenLiP3 database, respectively. And a protein-protein interaction (PPI) network of DE mRNAs was constructed with STRING and Cytoscape to identify hub genes. The genetic alterations, neighboring gene networks, expression levels, and the poor prognosis of hub genes were investigated in cBioPortal, Oncomine, and Human Protein Atlas databases and Kaplan-Meier plotter, respectively. A total of 10 DE miRNAs and 33 DEGs were identified. The regulatory network contained 26 circRNAs, 10 miRNAs, and 1459 mRNAs. Functional enrichment analysis revealed that the selected 33 DEGs were involved in negative regulation of fat cell differentiation, response to wounding, extracellular matrix- (ECM-) receptor interaction, and regulation of cell growth pathways. THBS1, FN1, CALM1, COL4A1, CTGF, and IGFBP5 were selected as inflammation-related hub genes of GC in the PPI network. The genetic alterations in these hub genes were related to amplification and missense mutations. Furthermore, the genes RYR2, ERBB2, PI3KCA, and HELZ2 were connected to hub genes in this study. The hub gene levels in clinical specimens were markedly upregulated in GC tissues and correlated with poor overall survival (OS). Our results suggest that THBS1, FN1, CALM1, COL4A1, CTGF, and IGFBP5 were associated with the pathogenesis of gastric carcinogenesis and may serve as biomarkers and inflammation-related targets for GC.

## 1. Introduction

Gastric cancer (GC) is one of the most common gastrointestinal malignancies in the clinic; in 2015, 1,313,000 people were diagnosed with GC, and 813,000 people died from it [1–9]. GC displays high heterogeneity with respect to histopathological and epidemiological characteristics [1, 10] and can be divided into proximal nondiffuse, diffuse, and distal nondiffuse subtypes [1, 11]. Gastric adenocarcinoma (GA) is the primary pathological type associated with environmental factors, including a high-salt diet, infectious agents, and smoking, and this form of GC is characterized by ease of invasion and metastasis and a very low early diagnosis rate [5, 12]. Despite the tremendous efforts that have been paid in the diagnosis of GC, together with improvements in surgical techniques and targeted chemotherapy in recent years, the prognosis of patients with GC remains unsatisfactory due to the prevalence of diagnosis of the disease at advanced stages that are often accompanied by lymphatic metastasis, which limits successful therapeutic strategies [7]. Additionally, cancer-related systemic inflammation is recognized as a flag sign of cancer-related cachexia development and progression, which results in an inappropriate systemic reaction, including fevers, weight loss, and sweats [13]. Therefore, further exploration of appropriate molecular biomarkers for early diagnosis and identification of potential inflammation-related targets of GC are urgently needed to expand the promising strategies to enhance the therapeutic efficacy and clinical prognosis in patients with GA.

Circular RNA (circRNA) is a novel type of endogenous, noncoding RNA, which is also widely expressed in different species and has been demonstrated to belong to a class of RNAs with tissue-/developmental-stage specificity, making them potential diagnostic and prognostic biomarkers [3, 14]. MicroRNAs (miRNAs) are a special type of small noncoding RNAs comprising approximately 21 nucleotides that bind to the 3′-untranslated regions (3′-UTRs) of target messenger RNAs (mRNAs) and leading to mRNA degradation or blocking of translation, which contribute to the central regulation of cell proliferation, differentiation, and apoptosis [9, 15, 16]. Recent studies have revealed that the abundance and evolutionary conservation of circRNAs play significant effects in regulating cancer progression-related processes such as metastasis, apoptosis, and invasion and that circRNAs primarily function as miRNA sponges, thereby relieving miRNA-mediated target repression. For instance, circRNA-ZFR serves as a sponge of miR-130a/miR-107 and modulates PTEN expression, resulting in the suppression of GC cell proliferation and the promotion of apoptosis [17]. Another report by Zhang et al. showed that circRNA-LARP4 inhibits cell proliferation and invasion in GC by sequestering miR-424-5p and regulating LATS1 expression [18]. Besides, it has been reported that knockdown of ciRS-133 reduced cancer cachexia by activating PRDM16 and suppressing miR-133 in tumor-implanted mice [19]. Although several circRNAs have been identified to participate in the pathogenesis of GC, it is still necessary to conduct a more comprehensive analysis of circRNA-miRNA-mRNA regulatory networks and to identify potential inflammation-related targets in GC.

**Figure 1:**
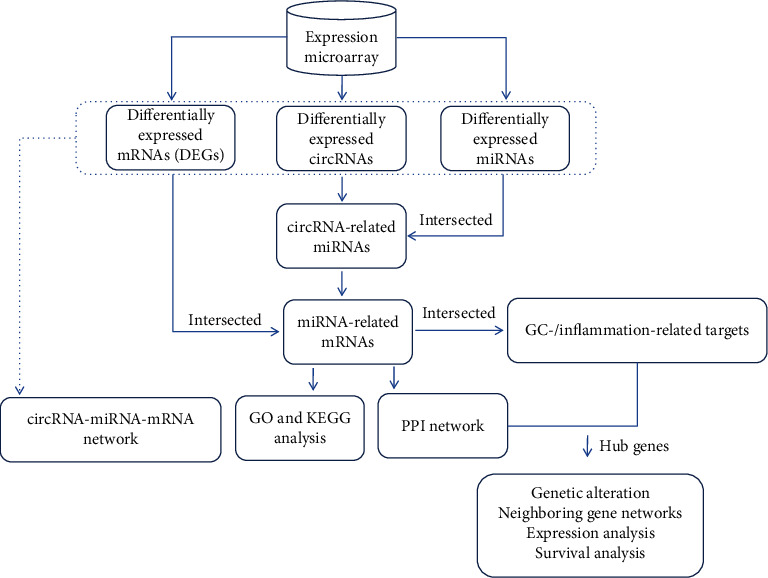
Workflow for the identification and analysis of hub genes in gastric adenocarcinoma.

In this study, we performed an integrated analysis of circRNA expression profiles in GC from the Gene Expression Omnibus (GEO) database. miRNAs and mRNAs from GEO datasets were employed to distinguish the circRNA-related dysregulated miRNAs and the miRNA-related dysregulated mRNAs, and then, a circRNA/miRNA/mRNA network was constructed to elucidate the relationships among differentially expressed (DE) circRNAs, miRNAs, and mRNAs. >GO (Gene Ontology) and KEGG (Kyoto Encyclopedia of Genes and Genomes) pathway enrichment analysis revealed the potential biological functions of selected miRNA target genes (DEGs). Besides, the inflammation-/GC-related targets were collected in the GeneCards and GenLiP3 database, respectively. Then, a PPI network of the selected DE mRNAs was constructed to identify the inflammation-related hub genes based on the value of the overall degree, node betweenness, and closeness through network topology calculations. Furthermore, genetic alterations and neighboring gene networks were investigated by cBioPortal, and the expression transcriptional level of hub genes was explored using Oncomine databases with subsequent validation using the Human Protein Atlas. Finally, survival analysis of hub genes was performed using the Kaplan-Meier plotter. These findings may enable us to identify novel diagnostic or prognostic biomarkers and suggested that THBS1, FN1, CALM1, COL4A1, CTGF, and IGFBP5 might be inflammation-related candidate targets for GC. The workflow of the current study is shown in Figure 1.

## 2. Materials and Methods

### 2.1. Microarray Data

We chose the circRNA expression profile GSE83521, the miRNA expression profile GSE78091, and the mRNA expression profile GSE33651 from the GEO (http://www.ncbi.nlm.nih.gov/gds/) database. GSE83521, which was based on Agilent GPL19978 (Agilent-069978 Arraystar Human CircRNA Microarray V1), includes 6 tumors and 6 adjacent normal mucosal tissues. GSE78091, which was based on Agilent GPL21439 (miRCURY LNA microRNA Array), includes 3 cancer tissues and 3 normal gastric mucosal tissues. The GSE33651 based on the platform of GPL2895 (Agilent, USA), including 40 gastric tumor tissue samples and 12 normal gastric tissue samples.

### 2.2. Data Processing

The raw data of microarray datasets were preprocessed via background correction and normalization. The box plot is a convenient way to quickly visualize the distribution of data, and we used box plots to examine and compare the distributions of expression profiles of samples after normalization. Hierarchical clustering, the most widely used clustering technique, allows us to observe the relationships between samples, and we performed a cluster analysis based on “All Targets Value-CircRNAs” in this study. DE circRNAs, miRNAs, and mRNAs were identified using GEO2R (https://www.ncbi.nlm.nih.gov/geo/geo2r/), an interactive online tool that permits users to compare two or more groups of samples to find out genes that are DE across experimental conditions [20]. ∣log_2_fold change (FC) | ≥2.0 and *P* value < 0.05 were used as the threshold criteria for the DE circRNAs, miRNAs, and mRNAs.

### 2.3. Construction of the circRNA-miRNA-mRNA Regulatory Network

DE circRNAs contain corresponding miRNA-binding sites and act as miRNA sponges. To further explore this connection, we predicted the interactions between the miRNAs and circRNAs by using the Circular RNA Interactome (http://circinteractome.nia.nih.gov) database [21]. Then, the circRNA-related miRNAs and DE miRNAs were intersected. miRNAs play important roles in a variety of diseases and regulate the expression of oncogenes and tumor suppressors. The regulatory relationships between miRNAs and mRNA were predicted using the miRTarBase database (http://miRTarBase.mbc.nctu.edu.tw/) [22]. Subsequently, the miRNA-related mRNAs and the DE mRNAs were also intersected to identify the selected DEGs by using Venny 2.1 (https://bioinfogp.cnb.csic.es/tools/venny/index.html). Finally, the DE circRNAs, predicted miRNAs, and mRNAs were added in a circRNA/miRNA/mRNA network. Moreover, the regulatory network was visualized with Cytoscape 3.4.0 (http://cytoscape.org/).

### 2.4. Functional Enrichment Analysis

GO and KEGG pathway enrichment analysis of selected DEGs was carried out using the DAVID (http://www.david.abcc.ncifcrf.gov/) database. GO terms (biological processes, cellular components, and molecular functions) and KEGG pathways with *P* < 0.05 were further analyzed. The enriched GO terms of selected DEGs were ranked by the enrichment scores (−log_10_ (*P* value)).

### 2.5. Identification of GC and Inflammation-Related Targets

The GC-related targets and inflammation-related targets were collected from the GeneCards (https://www.genecards.org/) [23] and GenCLiP 3 database (http://ci.smu.edu.cn/genclip3/analysis.php), respectively. GenCLiP 3, a web server, is enhancing the analysis of human gene functions and regulatory networks from PubMed based on cooccurrences and natural language processing [24]. In brief, the keywords “inflammation” and “gastric cancer” were used in the GeneCards and GenCLiP 3 database to search for GC-/inflammation-related targets, respectively. Next, the inflammation-related targets of GC were analyzed with Draw Venn Diagrams (http://bioinformatics.psb.ugent.be/webtools/Venn/).

### 2.6. PPI Network and Hub Gene Identification

The associations of the selected DEGs were analyzed using STRING (http://string-db.org/) [25], which provides both predicted and experimental PPI interaction information, and a PPI network was then constructed, which was visualized with Cytoscape software. Required confidence (combined score) ≥ 0.4 was used as the threshold criterion. Subsequently, to further analyze the more significant interactions of hub genes in the PPI network, we calculated the network topology of node degree, node betweenness, and closeness value using the Cytoscape plug-in Network Analyzer.

### 2.7. Exploring Cancer Genomics Data of Hub Genes Using cBioPortal

cBioPortal (http://cbioportal.org), an internet resource platform, is making complex cancer genomic profiles accessible to researchers and clinicians without the need for bioinformatics expertise [26, 27]. In the present study, we used cBioPortal to investigate candidate hub genes via stomach adenocarcinoma studies including 393 samples available in the database (The Cancer Genome Atlas (TCGA), Provisional). The results of the genomics datasets are shown through a concise and compact graphical summary of genomic alterations in multiple genes as heat maps, as well as multiple visualization networks of hub genes that are altered in cancer.

### 2.8. Oncomine Analysis and Validation

The publicly available online cancer microarray database Oncomine (http://www.oncomine.com), a collection of cancer microarray datasets with a comprehensive data-mining platform [28], can facilitate discovery from genome-wide expression analyses. Oncomine was chosen to explore the expression of hub genes in GC tissues compared with those in normal tissues. We chose to filter cancer vs. normal (analysis type), gastric adenocarcinoma (cancer type), clinical specimen (sample type), and mRNA (data type) to investigate the clinical significance in GC. Derrico gastric datasets were selected because they were established based on mRNA levels and contain larger numbers of samples (*n* > 50). In Derrico datasets, the thresholds for significance were 2-fold change, *P* value = 1*E*-4, and top 10% gene rank. The minimum, 10^th^, 25^th^, 75^th^, 90^th^, and maximum percentile data of each hub genes in both GC and normal tissues were plotted. Furthermore, the Human Protein Atlas (http://www.proteinatlas.org) was used to validate the immunohistochemistry of candidate hub genes. The direct links to these images in the Human Protein Atlas are as follows: https://www.proteinatlas.org/ENSG00000137801-THBS1/tissue/stomach#img (thrombospondin 1 (THBS1) in normal tissue); https://www.proteinatlas.org/ENSG00000137801-THBS1/pathology/tissue/stomach+cancer#img (THBS1 in tumor tissue); https://www.proteinatlas.org/ENSG00000115414-FN1/tissue/stomach#img (fibronectin 1 (FN1) in normal tissue); https://www.proteinatlas.org/ENSG00000115414-FN1/pathology/tissue/stomach+cancer#img (FN1 in tissue); https://www.proteinatlas.org/ENSG00000198668-CALM1/tissue/stomach#img (calmodulin-1 (CALM1) in normal tissue); https://www.proteinatlas.org/ENSG00000198668-CALM1/pathology/tissue/stomach+cancer#img (CALM1 in tumor tissue); https://www.proteinatlas.org/ENSG00000187498-COL4A1/tissue/stomach#img (collagen, type IV, alpha 1 (COL4A1) in normal tissue); https://www.proteinatlas.org/ENSG00000187498-COL4A1/pathology/tissue/stomach+cancer#img (COL4A1 in tumor tissue); https://www.proteinatlas.org/ENSG00000118523-CTGF/tissue/stomach#img (connective tissue growth factor (CTGF) in normal tissue); and https://www.proteinatlas.org/ENSG00000118523-CTGF/pathology/tissue/stomach+cancer#img (CTGF in tumor tissue).

### 2.9. Kaplan-Meier Plotter

The prognostic value of hub gene mRNA transcription level was measured using the Kaplan-Meier plotter (http://www.kmplot.com), an online open database that consists of gene expression profiles and survival information for 5,143 breast cancer, 1,816 ovarian cancer, 2,437 lung cancer, and 1,065 GC patients with mean follow-ups of 69, 40, 49, and 33 months, respectively. To evaluate the overall survival (OS) of patients with GC, we separated individuals into two groups based on median gene expression (high vs. low) and then validated their Kaplan-Meier survival curves. Hazard ratios (HRs) with 95% confidence intervals (CIs) and log-rank *P* values were calculated to evaluate the associations of gene expression with survival, and the number-at-risk values were displayed below the curves.

## 3. Results

### 3.1. Screening of DE circRNAs, miRNAs, and mRNA in GC

**Figure 2:**
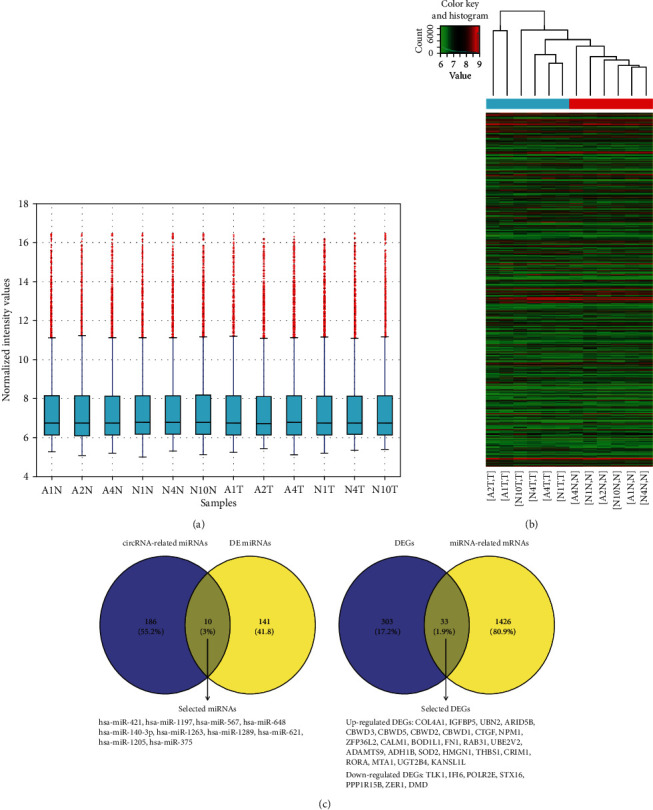
DE circRNAs in tumor tissues and adjacent normal tissues from GC patients, screening DE miRNAs and DE mRNAs, and visualization of the circRNA-miRNA-mRNA regulatory network. (a) The box plot shows variations in circRNA expression. (b) The heat map shows DE circRNAs in 6 tumors (T) and 6 normal (N) tissues. Gene expression profiles are shown in rows. “Red” suggests a high relative expression, and “green” indicates low relative expression. (c) Based on DE miRNAs and circRNA-related miRNAs, Venn diagrams were used to select the overlapping 10 miRNAs; based on DE mRNAs and miRNA-related mRNAs, Venn diagrams were used to select the overlapping 33 mRNAs.

The circRNA expression profile of GSE83521 was deposited by Zhang Y from Southern Medical University, Nanfang Hospital. This dataset has 6 tumor tissue samples and 6 normal mucosal tissues. As shown in the box plot (Figure 2(a)), the median intensity values in different samples were almost similar after normalization, which showed optimal standardization. Next, the map showed the dissimilarity of circRNA expression patterns among tissue samples (Figure 2(b)). And a total of 26 DE circRNAs were identified after the analysis of GSE83521 (Tables S1 and S2), of which 25 were upregulated and 1 was downregulated. A total of 151 DE miRNAs were detected for GSE78091 (Table S3), of which 148 were upregulated and 3 were downregulated. Furthermore, a total of 336 DE mRNAs were detected after the analysis of GSE33651 (Table S4), of which 254 were upregulated and 82 were downregulated.

### 3.2. Construction of the ceRNA Network

Increasing numbers of reports have shown that by acting as competing for endogenous RNAs (ceRNAs), circRNAs can compete with miRNAs to influence the stability of target mRNAs or their translation. In this study, 418 interactions were obtained between 26 DE circRNAs and 196 miRNAs (Table S5). Ten miRNAs were selected after the intersection of DE miRNAs and circRNA-related miRNAs (Figure 2(c)). The target genes of the 10 selected miRNAs were predicted using miRTarBase, and 1569 interactions were detected between these 10 selected miRNAs and 1459 miRNA-related mRNAs (Table S6). Then, thirty-three DEGs were screened after the intersection between DEGs and miRNA-related mRNAs (Figure 2(c)).

**Figure 3:**
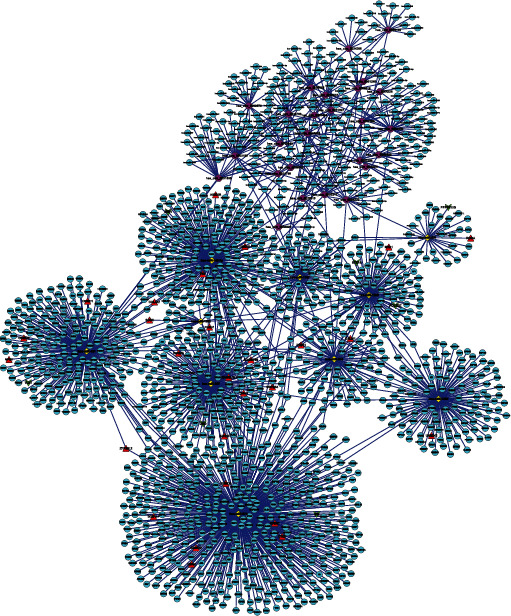
The circRNA-miRNA-mRNA regulatory network in GC. The circular nodes in blue represent mRNAs; the diamond-shaped nodes in yellow represent miRNAs, and the octagonal nodes in purple represent circRNAs. The triangular nodes in red indicate upregulated DE mRNAs, and the V nodes in green indicate downregulated DE mRNAs.

More importantly, a ceRNA network was established to uncover the connections between circRNAs, miRNAs, and mRNAs in this study. This network contained 401 circRNA-miRNA pairs and 1568 miRNA-mRNA pairs, including 26 circRNAs, 10 miRNAs, and 1459 mRNAs (Figure 3). Subsequently, the selected DEGs were analyzed with respect to their potential bioinformatics information.

### 3.3. Functional Enrichment Analysis of Selected DEGs

**Figure 4:**
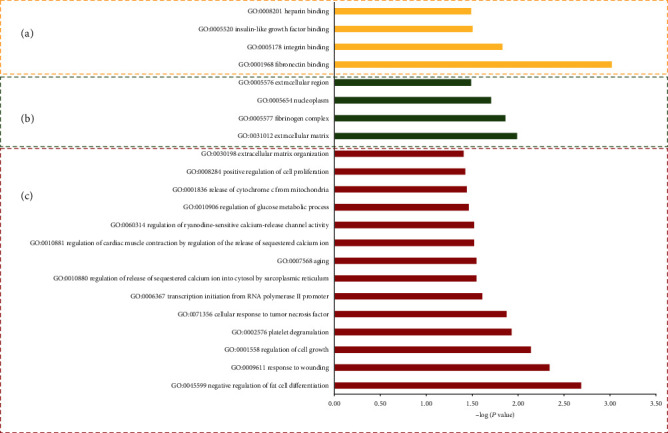
The GO term enrichment analysis on 33 selected DEGs in GC. (a) Thin yellow bars represent molecular function terms. (b) Green bars represent cell component terms. (c) Red bars represent biological processes.

The selected 33 DEGs of the associated circRNAs were used to investigate the GO terms and KEGG pathways by using DAVID. In GO analysis, all enriched terms for the DEGs were ranked by enrichment score (-log_10_(*P* value)) as shown in Figure 4. The most significantly enriched GO term regarding molecular functions (Figure 4(a)) was fibronectin binding (GO:0001968, *P* = 9.66*E* − 04). The most significantly enriched GO terms with respect to cellular components (Figure 4(b)) were extracellular matrix (ECM) (GO:0031012, *P* = 0.010291067), fibrinogen complex (GO:0005577, *P* = 0.013746239), and nucleoplasm (GO:0005654, *P* = 0.019723458), and the top 3 most significantly enriched GO terms with respect to biological process (Figure 4(C)) were negative regulation of fat cell differentiation (GO:0045599, *P* = 0.002060324), response to wounding (GO:0009611, *P* = 0.004577343), and regulation of cell growth (GO:0001558, *P* = 0.007282945). Moreover, the ECM-receptor interaction pathway (hsa04512, *P* = 0.011170014) associated with the genes COL4A1, THBS1, and FN1 was represented as the most enriched pathway.

### 3.4. Analysis of the PPI Network

**Figure 5:**
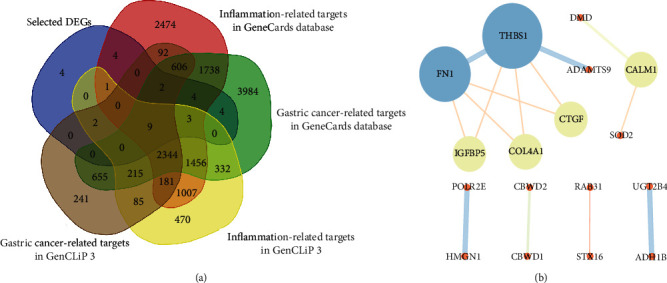
The identification of inflammation-related targets in GC by Venn diagram and PPI analysis: (a) Venn diagrams of the inflammation-related targets between the selected DEGs and the integrated GC-/inflammation-related targets in the GeneCards and GenCLiP 3 database; (b) the PPI network of 33 coexisting DEGs. Nodes represent genes, with the degree shown by the size and bright colors, and edges represent interactions between two genes, with the combined scores displayed by the size and bright colors.

With the retrieving of the GeneCards and GenCLiP 3 database, we finally obtained a total of 15,784 and 16,027 genes related to GC and inflammation, respectively (Tables S7 and S8). After intersection with selected DEGs, we found that 9 DEGs may be associated with the inflammation in the GC, including SOD2, FN1, THBS1, MTA1, NPM1, IGFBP5, COL4A1, RORA, and ADH1B (Figure 5(a)). To explore the relationships among the 33 selected DEGs, we constructed the PPI networks for genes using STRING and then visualized in Cytoscape. Degree denotes the numbers of proteins interacting with a specific protein, and a node with a high degree is deemed a hub node [29]. Hub genes are obtained by analyzing the connectivity degrees, betweenness, and closeness of the nodes in PPI networks. As presented in Figure 5(b), the genes UBN2, ARID5B, CBWD3, CBWD5, NPM1, ZFP36L2, BOD1L1, UBE2V2, CRIM1, RORA, MTA1, KANSL1L, TLK1, IFI6, PPP1R15B, and ZER1 with no interactions were removed in the STRING database. The obtained PPI networks consisted of 17 nodes and 14 edges, with 6 central node genes identified using degree ≥ 2, and the most significant 6 node degree genes for subsequent study were THBS1 (degree = 5, closeness centrality = 1), FN1 (degree = 4, closeness centrality = 0.83), CALM1 (degree = 2, closeness centrality = 1), COL4A1 (degree = 2, closeness centrality = 0.625), CTGF (degree = 2, closeness centrality = 0.625), and insulin-like growth factor-binding protein 5 (IGFBP5, degree = 2, closeness centrality = 0.625).

### 3.5. Genetic Alterations in Hub Genes in GC

**Figure 6:**
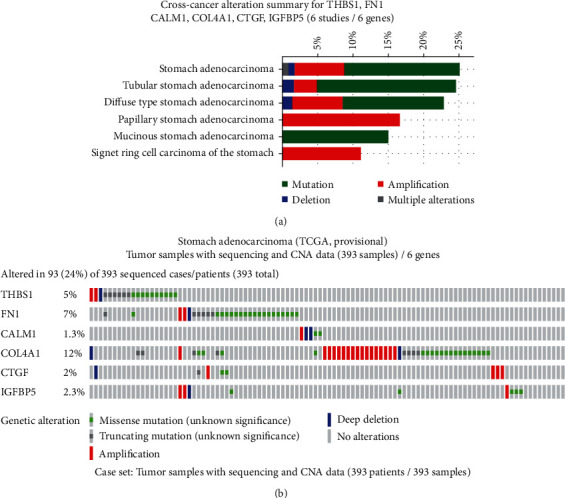
Exploring genetic alterations in THBS1, FN1, CALM1, COL4A1, CTGF, and IGFBP5 in GC by cBioPortal: (a) summary of changes within THBS1, FN1, CALM1, COL4A1, CTGF, and IGFBP5 genes in genomics datasets available for stomach adenocarcinoma (TCGA, Provisional) studies; (b) OncoPrint: a visual summary of alterations across a set of stomach adenocarcinoma samples (data are taken from TCGA Data Portal) based on a query of the 6 genes (THBS1, FN1, CALM1, COL4A1, CTGF, and IGFBP5). Distinct genomic alterations are summarized, color-coded, and presented as percent changes in particularly affected genes in individual tumor samples. Each row represents a gene, and each column represents a tumor sample. Green squares indicate missense mutations; black represents truncating mutations; red bars designate gene amplifications; blue bars represent deep deletions, and grey signifies no alterations.

Combining the results of functional analysis, the aforementioned Venn Diagrams and PPI network indicated that THBS1, FN1, CALM1, COL4A1, CTGF, and IGFBP5 may play an important role in GC as the inflammation-related targets. We further verified the genetic alterations in the selected 6 hub genes in GC patients by using the cBioPortal database. As illustrated in Figure 6, alterations ranging from 11.11% to 25.11% were found for the gene sets submitted for analysis (Figure 6(a)). cBioPortal complements existing tools, including TCGA. Multiple genetic alterations observed across each set of tumor samples from TCGA Data Portal are presented using OncoPrint to highlight the most pronounced genomic changes. The results showed that 96 cases (24%) had an alteration in at least one of the 6 queried genes. Most alterations in the THBS1 and FN1 genes were classified as missense mutations, along with a few cases of truncating mutations, amplifications, and deep deletions (Figure 6(b)). A majority of alterations in the COL4A1 gene occurred by amplification and missense mutations, with a few cases of partial missense mutations and deep deletions (Figure 6(b)). For the CALM1, CTGF, and IGFBP5 genes, missense mutations, truncating mutations, amplifications, and deep deletions occurred in select cases (Figure 6(b)).

**Figure 7:**
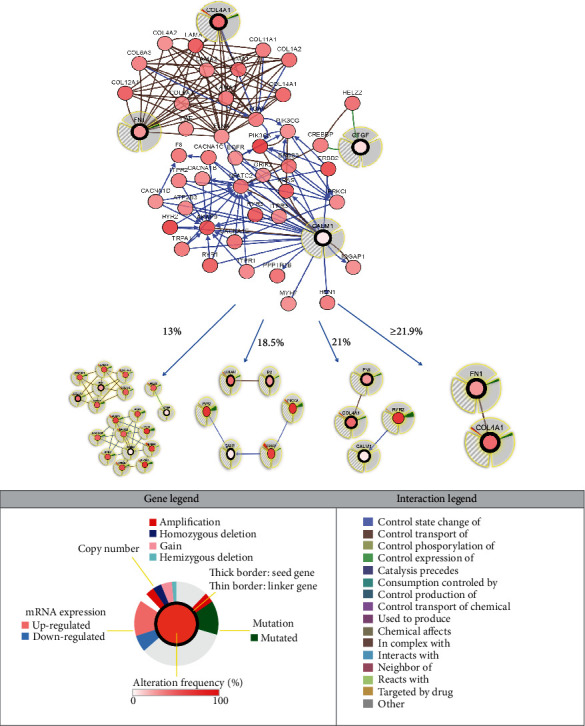
Display of neighboring genes connected to THBS1, FN1, CALM1, COL4A1, CTGF, and IGFBP5 in GC. THBS1, FN1, CALM1, COL4A1, CTGF, and IGFBP5 were used as seed genes (indicated with thick black borders) to automatically harvest all other genes identified as altered in GC. Multidimensional genomic details are shown for COL4A1, FN1, CTGF, and CALM1 seed genes. Dark red indicates an increased frequency of alterations (defined by mutation, copy number amplification, or homozygous deletion) in GC. Shown in the figure are the full and pruned networks containing all or part neighbors of all query genes generated; the neighboring genes connected to COL4A1, FN1, CTGF, and CALM1 are filtered by alteration (%).

The potential of complexity, as well as the variability of differences in interactions between hub genes in GC samples, was studied from TCGA Data Portal with multiple visualization networks generated. To identify potential interactive analysis in GC, we used THBS1, FN1, CALM1, COL4A1, CTGF, and IGFBP5 as core nodes for the network view and investigated the resulting altered networks of interest. cBioPortal was used to construct a network containing FN1, CALM1, COL4A1, and CTGF in addition to THBS1 and IGFBP5 (Figure 7). The interactions between FN1 and COL4A1 were identified using a cutoff of ≥21.9% alteration. The interactions between CALM1 and RYR2 hub genes were revealed using a cutoff of 21% alteration. ERBB2 and PIK3CA were observed with a cutoff of 18.5% alteration, and the interactions between CTGF and HELZ2 hub genes appeared when the cutoff was changed to 13% alteration (Figure 7). The results of interactive network analysis provide new perspectives on the role of hub genes, along with RYR2, ERBB2, PIK3CA, and HELZ2, with respect to the development of GC by querying multidimensional cancer genomics data in cBioPortal.

### 3.6. Expression and Validation of Hub Genes in GC

**Figure 8:**
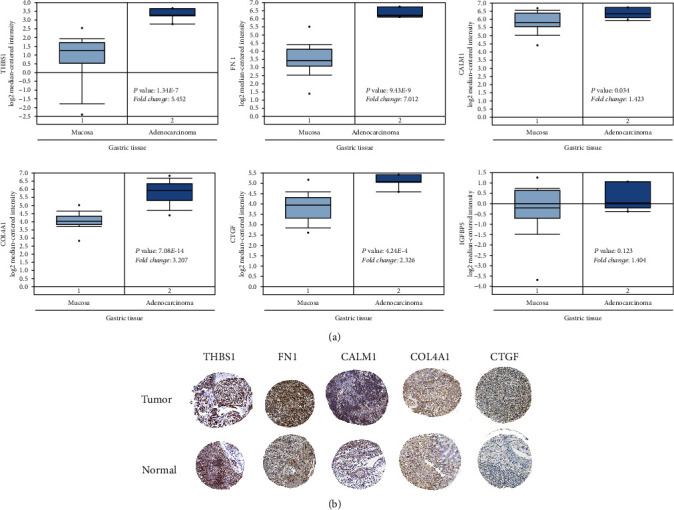
mRNA and protein expression levels of THBS1, FN1, CALM1, COL4A1, CTGF, and IGFBP5 for clinical significance in GC: (a) Oncomine data mining showing that THBS1, FN1, CALM1, COL4A1, CTGF, and IGFBP5 mRNA expression levels in Derrico datasets differ between normal mucosa and gastric adenocarcinoma tissues; (b) protein levels of THBS1 in normal tissue (staining: high; intensity: strong; quantity: >75%). Protein levels of FBXO5 in tumor tissue (staining: high; intensity: strong; quantity: >75%). Protein levels of FN1 in normal tissue (staining: low; intensity: moderate; quantity: <25%). Protein levels of FN1 in tumor tissue (staining: medium; intensity: moderate; quantity: 75%-25%). Protein levels of CALM1 in normal tissue (staining: medium; intensity: moderate; quantity: 75%-25%). Protein levels of CALM1 in tumor tissue (staining: medium; intensity: moderate; quantity: 75%-25%). Protein levels of COL4A1 in normal tissue (staining: not detectable; intensity: negative; quantity: negative). Protein levels of COL4A1 in tumor tissue (staining: medium; intensity: moderate; quantity: 75%-25%). Protein levels of CTGF in normal tissue (staining: low; intensity: moderate; quantity: rare). Protein levels of CTGF in tumor tissue (staining: low; intensity: weak; quantity: >75%).

We also estimated the mRNA expression levels of the THBS1, FN1, CALM1, COL4A1, CTGF, and IGFBP5 in GC compared with those in normal tissues via the Oncomine database. In Derrico datasets, which has 69 samples, the mRNA levels separated the GC cases in Oncomine into intestinal, diffuse, and mixed types. As shown in Figure 8(a), THBS1, FN1, CALM1, COL4A1, CTGF, and IGFBP5 expression levels were markedly upregulated in mixed-type GC than in normal controls. Besides, the protein levels of these 5 genes (IGFBP5 is pending control and cancer tissue analysis) were observably higher in tumor tissues than in normal tissues, according to the data obtained from the Human Protein Atlas (Figure 8(b)).

### 3.7. Survival Analysis

**Figure 9:**
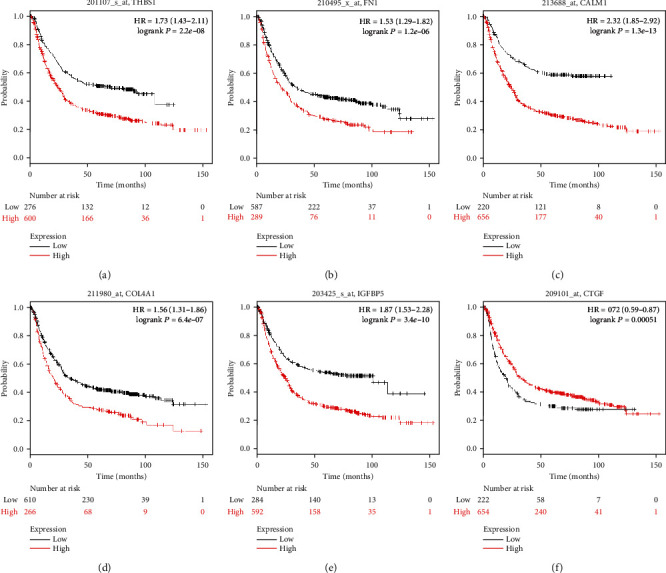
Prognostic curve for the six hub genes. The prognostic significance of the hub genes in patients with GC determined using the Kaplan-Meier plotter. Affymetrix IDs (a) 201107_s_at, (b) 210495_x_at, (c) 213688_at, (d) 211980_at, (e) 203425_s_at, and (f) 209101_at represent THBS1, FN1, CALM1, COL4A1, IGFBP5, and CTGF, respectively. Red lines represent patients with high gene expression, and black lines represent patients with low gene expression.

The Kaplan-Meier plotter was applied to predict the prognostic value of the 6 identified hub genes. According to the results, high expression levels of THBS1, FN1, CALM1, COL4A1, and IGFBP5 were closely related to the poor OS in GC patients (*P* < 0.05) (Figures 9(a)–9(e)). In contrast, low expression of CTGF was associated with poor OS in GC patients (*P* < 0.05, Figure 9(f)).

## 4. Discussion

GC still remains the commonest human malignancy in the world [30, 31]. Nevertheless, surgical resection is the only possible curative therapy for GC. In fact, approximately 60% of GC patients are diagnosed with metastatic and locally advanced, which results in poor prognosis due to deficiency of early detection and the loss of the opportunity for curative resection [30]. Additionally, inflammation has long been recognized to play a crucial role in the pathogenesis of cancer [13, 32]. Hence, the identification of novel diagnostic markers and inflammation-related targets and the elucidation of the underlying mechanisms of GC onset and progression have become major topics in GC research. In the current study, the circRNA expression profile GSE83521, the miRNA expression profile GSE78091, and the mRNA expression profile GSE33651 were acquired from the GEO database to reanalyze DE circRNAs, miRNAs, and mRNAs between GC tissues and normal tissues using bioinformatics method to identify novel diagnostic markers and explore the inflammation-related targets for GC. In total, 26 DE circRNAs including 25 upregulated and 1 downregulated circRNAs, 151 DE miRNAs including 148 upregulated and 3 downregulated miRNAs, and 336 DE mRNAs including 254 upregulated and 82 downregulated mRNAs with *P* < 0.05 and ∣log_2_FC | ≥2.0 were screened; additionally, 33 DEGs were selected for further study after DE mRNA and miRNA-related mRNA intersection.

The results of functional enrichment analysis showed that the 33 DEGs were associated with the negative regulation of fat cell differentiation, response to wounding, and regulation of cell growth. Compared with the study by Gu et al. [3], which showed that the p53 signalling pathway and the Hippo signalling pathway were significantly enriched in GC, while the KEGG results indicated that the ECM-receptor interaction pathway might be involved in the development of GC in this study. Meanwhile, the results are consistent with the previous study [33]. The ECM-receptor interaction pathway has been identified in multiple cancers, and it includes “cell adhesion molecules (CAMs)” and “cell cycle” pathways [34]. Imbalances in these pathways lead to the detachment of cells from the ECM and thus enhanced metastasis, suggesting an essential role of this pathway in cancer biology [34]

circRNAs are a class of endogenous noncoding RNAs that primarily serve as miRNA sponges to regulate gene expression and are reported to play important roles in many malignant phenotypes, including the cell cycle, apoptosis, vascularization, invasion, and metastasis [35–38]. In addition, circRNAs may serve as a novel and stable biomarkers for the diagnosis of GC. Li et al. demonstrated that hsa_circ_002059, a typical circRNA, is significantly downregulated in GC tissues compared with that in paired adjacent nontumor tissues, indicating its potential as a novel and stable biomarker for the diagnosis of GC [39]. Another study by Huang et al. found that the circRNA hsa_circ_0000745 can serve as a diagnostic marker for GC [40]. Gu et al. [3] also revealed that CCND2 might be regulated by hsa_circRNA_105039 and hsa_cirRNA_104682 through hsa-miR-15a-5p. Compared with previous studies, we found that the hsa_circ_0001866/hsa_circ_0003192-hsa-miR-421-COL4A1, hsa_circ_0055521-hsa-miR-567-THBS1/IGFBP5, hsa_circ_0005217/hsa_circ_0004370-hsa-miR-648-THBS1/CALM1, hsa_circ_0007094/hsa_circ_0013048/hsa_circ_0008615/hsa_circ_0002570/hsa_circ_0001789-hsa-miR-140-3p-FN1, hsa_circ_0004339/hsa_circ_0007094/hsa_circ_0007613-hsa-miR-1205-THBS1, and hsa_circ_0007404/hsa_circ_0051246/hsa_circ_0045602/hsa_circ_0031027-hsa-miR-375-CTGF axes may play central roles in regulating the development of GC.

Further, the mechanisms underlying of the molecular targets in GC remains to be elucidated. In this study, THBS1, FN1, CALM1, COL4A1, CTGF, and IGFBP5 might be identified as the hub genes associated with inflammation in GC from the PPI network. We also identified missense mutations and amplifications as the primary genetic alterations. Additionally, survival analysis showed that high mRNA expression of THBS1, FN1, CALM1, COL4A1, and IGFBP5 was correlated with poor OS in GC patients; in contrast, high mRNA expression of CTGF was associated with poor OS in GC patients.

Thrombospondins are a family of homologous proteins that regulate cellular phenotypes and extracellular structures for tissue genesis and remodelling [41]. The first member to be identified, THBS1, is an extracellular glycoprotein that plays multifunctional roles in the cell-matrix and cell-cell interactions, angiogenesis, and tumor progression [42–45]. THBS1 expression has been correlated with tumor angiogenesis, tumor growth, and metastasis [46]In GC, THBS1 may play a proangiogenic and proinflammatory role due to its positive correlation with vascular endothelial growth factor (VEGF), and elevated THBS1 expression levels have been related to tumor growth and lymph node metastasis in GC [47, 48]. The function of THBS1 remains controversial, and increasing numbers of cellular assays have indicated a role for THBS1 in cell invasion and migration; nevertheless, conflicting results have been obtained in different cell types. Notably, the pleiotropic nature of THBS1, which is a multimodular and multifunctional protein, depends on environmental conditions. We noted that the expression of THBS1 was higher in cancer tissues than in adjacent normal tissues in Derrico gastric datasets from the Oncomine database, which indicated that the results of the GSE33651 microarray were in agreement with the previous studies [43, 45].

FN1, a high molecular weight (~440 kDa) ECM cell-adhesive glycoprotein, is predominantly expressed in various cancer tissues but not in normal tissues [49] and involved in the ECM-receptor interaction, focal adhesion pathway, pathways in cancer, and the regulation of endothelial cell survival, proliferation, adhesion, migration, inflammation, and angiogenesis through the activation of the focal adhesion kinase (FAK) and downstream PI3K/Akt signalling pathways, as well as the activation of NF-*κ*B [50, 51]. Chen and Zheng and Zhang et al. reported that miRNA-200c binds to and inhibits the expression of FN1 to suppress the proliferation, migration, and invasion of GC cells [52, 53]. COL4A1, an essential component of the ECM that is also involved in the ECM-receptor interaction and focal adhesion pathway, has an essential role in angiogenesis, inflammation, and tumor progression [50]. Huang et al. demonstrated that COL4A1 is overexpressed in GC tissues and trastuzumab-resistant GC cells based on bioinformatics analysis [54], which is consistent with the expression data of COL4A1 in the GSE33651 and Oncomine gastric datasets.

CALM, a ubiquitous eukaryotic calcium-binding protein that transduces much of the calcium signal [55] and plays an important role in intercellular communication, cell movement, cell differentiation, cell proliferation, inflammation, and other physiological activities [56], is encoded by three nonallelic CALM genes (CALM1, CALM2, and CALM3). In the present study, the mRNA expression level of CALM1 was higher in GC tissues than in adjacent normal tissues. Hence, further studies of CALM1 in GC are necessary to clarify the role of CALM1 and its related molecular mechanisms in regulating physiological activities in GC.

Cyr61/CTGF/Nov (CCN) proteins are a family of matricellular proteins that consisted of an N-terminal secretory signal peptide and four structural modules and play pivotal context-dependent roles in many physiological and pathological processes according to tumor type, including inflammation [57]. CTGF (also known as CCN2) is involved in comprehensive regulatory processes in angiogenesis, chondrogenesis, osteogenesis, inflammation, fibrogenesis, diabetic nephropathy, and tumor development [58]. The Hippo signalling pathway is an emerging kinase cascade in gastrointestinal homeostasis and tumorigenesis. Kang et al. first revealed that CTGF is the key downstream effector for the oncogenic function of YAP1 in GC and that it is highly expressed in primary tumors [59]. Jiang et al. also demonstrated that high CCN2 expression is correlated with increased lymph node metastasis, enhanced peritoneal dissemination, and short five-year survival [60]; these findings are also consistent with the expression of CTGF in GSE33651 and Oncomine GC datasets, indicating a proliferation-promoting role of CTGF in cancer.

Insulin and insulin-like growth factors (IGFs) consist of three peptide ligands (INS, IGF1, and IGF2), three specific cell surface receptors (INSR, IGF1R, and IGF2R), and ten specific IGF-binding proteins (IGFBPs; IGFBP1-7, and IGF2BP1-3) [61, 62], which are considered to play important roles in hormonal regulation, inflammation, and signal transduction during energy metabolism and oncogenesis [62]. IGF activity is tightly regulated by a family of IGFBPs. IGFBP5, the most conserved member of the IGFBP family, is significantly upregulated during the differentiation of several key cell lineages and in human cancer and metastatic tissues. IGFBP5 plays several outstanding roles in carcinogenesis to regulate cell growth, migration, and invasion during the development of cancer [63], but its function in the progression of cancer is controversial. Accumulating evidence has revealed that IGFBP5 can suppress tumor growth and metastasis in various tissues and under different contexts, but IGFBP5 can also function as an oncogene, promoting metastasis in a context-dependent manner [64]. Furthermore, bladder cancer [62], colorectal cancer [65], and breast cancer [66] tissues also contain high levels of IGFBP5, but few studies have explored the expression and mechanism for IGFBP5 in human GC. Our previous study has revealed that IGFBP5 exhibits a distinctly different expression pattern in GC tissues based on GSE33651 and Oncomine GC datasets.

In summary, we conducted an integrated analysis of DE circRNAs, miRNAs, and mRNAs in GC and constructed a potential circRNA-miRNA-mRNA regulatory network. We identified THBS1, FN1, CALM1, COL4A1, CTGF, and IGFBP5 as potential inflammation-related targets for the treatment of gastric adenocarcinoma. Our bioinformatics analysis represents a useful strategy to explore malignancy from a new perspective. However, future studies are required to validate the mechanisms related to the potential target genes.

## Data Availability

The data used to support the findings of this study are available from the corresponding author upon request.

## Supplementary Materials

Supplementary 1 Table S1: circRNA expression profiling data.

Supplementary 2 Table S2: differentially expressed circRNAs between tumor and normal tissues.

Supplementary 3 Table S3: differentially expressed miRNAs between tumor and normal tissues.

Supplementary 4 Table S4: differentially expressed mRNAs between tumor and normal tissues.

Supplementary 5 Table S5: circRNA-related miRNAs.

Supplementary 6 Table S6: miRNA-related mRNAs.

Supplementary 7 Table S7: GC-related genes in the GeneCards and GenCLiP 3 database.

Supplementary 8 Table S8: inflammation-related genes in the GeneCards and GenCLiP 3 database.
